# Device-Level Modeling, Cross-Axis Analysis, and Optical Characterization of a Symmetric Triple-Layer MOEMS Accelerometer

**DOI:** 10.3390/mi17080984

**Published:** 2026-08-20

**Authors:** Pengfei Li, Shuang Wu, Wenhui Yan, Yujie Xiong, Jiaxin Sun, Chaoyue Shi, Haiyan Wang, Xiaoxu Wang, Qianbo Lu

**Affiliations:** 1 School of Marine Science and Technology, Northwestern Polytechnical University, 127 West Youyi Road, Xi’an 710072, China; pfcrazy@mail.nwpu.edu.cn (P.L.); hywang@nwpu.edu.cn (H.W.); 2 School of Automation, Northwestern Polytechnical University, 127 West Youyi Road, Xi’an 710072, China; 2021100718@mail.nwpu.edu.cn (W.Y.); s8520jx@163.com (J.S.); woyaofly1982@nwpu.edu.cn (X.W.); 3 State Key Laboratory of Flexible Electronics (LOFE), Institute of Flexible Electronics (IFE), Northwestern Polytechnical University, 127 West Youyi Road, Xi’an 710072, China; xiongyujie@mail.nwpu.edu.cn (Y.X.); 15373383517@163.com (C.S.)

**Keywords:** MOEMS accelerometer, MEMS inertial sensor, optical readout, wafer-level stacking, cross-axis coupling, acceleration noise, proof-mass enhancement

## Abstract

Enhancing the proof mass without enlarging the chip area or introducing structural asymmetry is a central challenge in the development of low-noise microelectromechanical system (MEMS) accelerometers. Here, we present a symmetric triple-layer MOEMS accelerometer and analyze its device-level sensitivity trade-off, cross-axis coupling behavior, and dynamic consistency between measurement and finite-element simulations. The proposed sensing element sandwiches one without-beam mass layer between two identical with-beam layers, thereby increasing the effective proof mass while preserving mirror symmetry. A lumped-parameter model is developed to explain the sensitivity trade-off among single-layer, asymmetric double-layer, and symmetric triple-layer configurations. Finite-element simulations are used to distinguish translational cross-axis coupling from rotational cross-axis coupling. The experimental characterization of one packaged triple-layer prototype demonstrates a mechanical sensitivity of 193.91 µm/(m/s^2^), a 10 min output RMS fluctuation of 1.81 µg, and a measured first-order resonant frequency of 11.23 Hz, in close agreement with the tolerance-included finite-element prediction of 11.40 Hz. The resonance bandwidth further yields an apparent package-level quality factor of approximately 374 under ambient pressure, providing additional characterization of the packaged device dynamics.

## 1. Introduction

Pushing the performance of MEMS accelerometers toward the sub-µg regime is fundamentally a challenge for noise reduction and sensitivity improvement [1,2,3]. The intrinsic noise source, mechanical–thermal noise, scales inversely with the three-fourths power of the proof mass but only with the fourth root of the beam stiffness [4]. For a given chip footprint, this scaling law makes mass enhancement a far more effective strategy than stiffness reduction for lowering the noise floor. The central difficulty lies in how to substantially increase the proof mass without violating the stringent constraints of the chip size, fabrication compatibility, and mechanical stability.

Existing mass enhancement techniques each face critical trade-offs. Expanding the proof mass area by shrinking other functional zones [5,6,7] limits beam design freedom and compromises sensitivity and bandwidth. Depositing high-density metals, such as gold or tungsten, onto the proof mass [8,9,10] yields a sizable mass increase but inevitably introduces high residual stress and thermal expansion mismatch with the silicon substrate, leading to warping, thermal drift, and long-term instability [11,12]. Silicon-on-insulator (SOI) and double-device-layer SOI approaches [13,14,15] can decouple the suspension-beam thickness from the bulk proof-mass thickness, enabling thick proof masses with well-controlled compliant beams; however, their structural configurations must be carefully designed to preserve symmetry and suppress cross-axis coupling.

Other MEMS accelerometers employ discrete high-density proof masses, such as tungsten, attached to compliant beams to increase inertial mass [16]. Such heterogeneous proof-mass integration can improve sensitivity, but it introduces additional material interfaces and fabrication/assembly considerations. Another category of methods is bonding-based stacking [17,18,19], which stacks multiple structural layers through wafer-level bonding to increase the structural thickness and mass. This strategy often employs direct bonding [20], which provides good mechanical strength and thermal stability but is typically conducted under high temperature and pressure, posing risks of thermal deformation for low-stiffness structures and creating challenges in residual-stress control.

In summary, there remains a need for a mass enhancement strategy for accelerometers that simultaneously provides low process-induced stress, structural symmetry, and compatibility with standard MEMS fabrication. This need is particularly acute in applications such as geophysical monitoring [21,22,23] and precision navigation [24,25], where sub-µg-level noise and stable low-drift operation are important.

Building on the previously reported wafer-level hybrid wet/dry etching and low-temperature bonding route [26], this study examines the device-level behavior of the symmetric triple-layer MOEMS accelerometer, including the lumped-parameter sensitivity trade-off, translational and rotational cross-axis responses, and measured/simulated resonant-frequency consistency.

The main contributions of this article are therefore threefold. First, a symmetric triple-layer MOEMS accelerometer architecture is analyzed using a lumped-parameter model that clarifies why mass enhancement and suspension stiffness must be considered together. Second, cross-axis behavior is separated into *z*-axis-induced parasitic translational displacement and *x*-axis-induced parasitic rotation, enabling a clearer interpretation of the role of mirror symmetry. Third, optical-readout-based characterization is used to evaluate the mechanical sensitivity and measured/simulated resonant-frequency consistency of the packaged prototype. A comparative discussion with representative MEMS/MOEMS inertial sensors is also provided to clarify the performance position and application relevance of the proposed architecture.

## 2. Materials and Methods

### 2.1. Device Architecture and Lumped-Parameter Model

**Figure 1:**
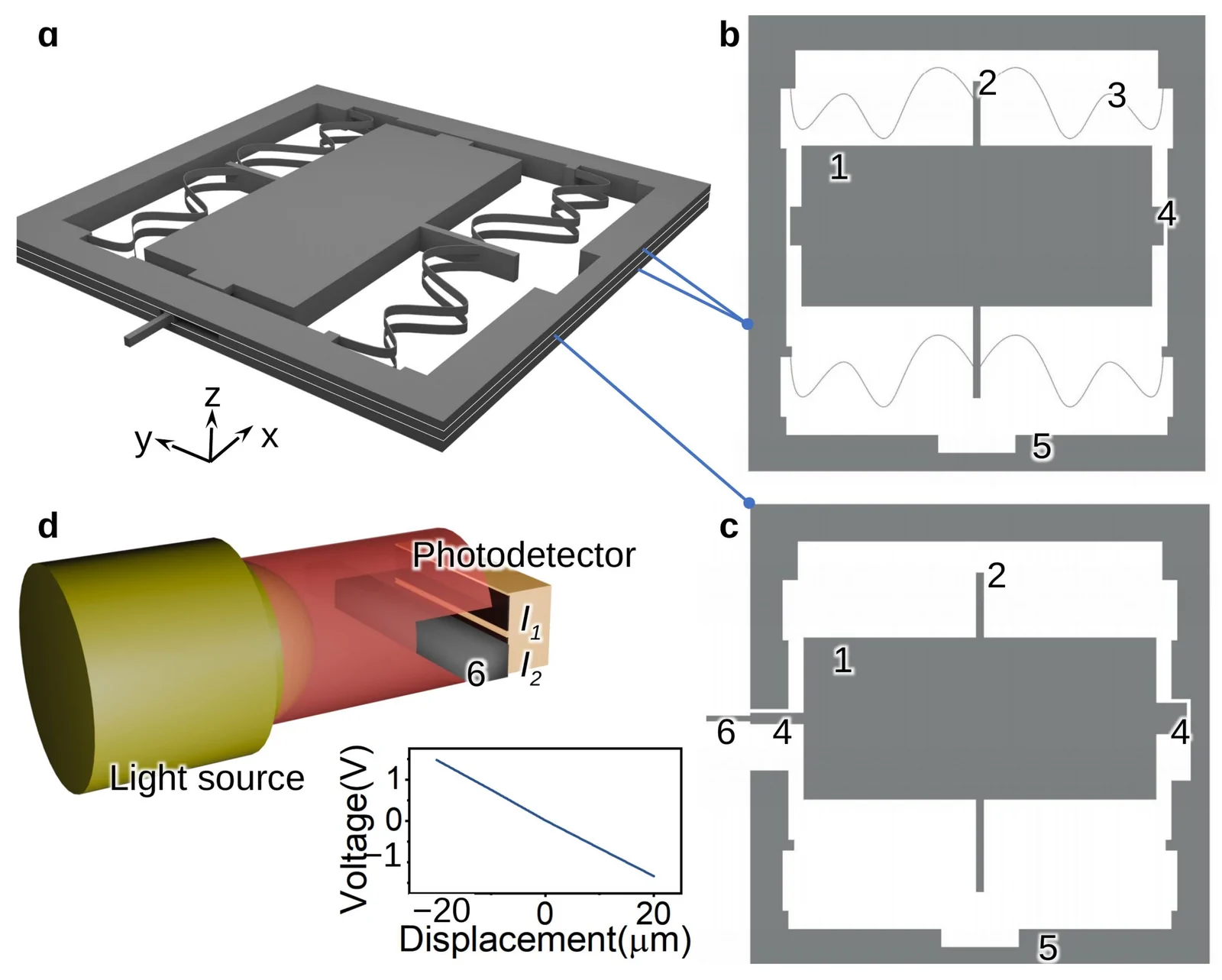
Schematic of the acceleration-sensing element. (**a**) Overall structure schematic; (**b**) with-beam layer, comprising (1) sensitive proof mass, (2) connecting rod, (3) freeform geometry beam, (4) stopper structure, and (5) frame; (**c**) without-beam layer, comprising (1) sensitive proof mass, (2) connecting rod, (4) stopper structure, (5) frame, and (6) shutter rod; (**d**) schematic of the optical displacement measurement, with an inset showing the output-voltage-versus-displacement curve.

The designed sensing element consists of three vertically stacked silicon-based structures, as shown in Figure 1a. The sensitive axis is the *y*-axis. From top to bottom, the structure comprises an upper with-beam layer (Figure 1b), a middle without-beam layer (Figure 1c), and a lower with-beam layer identical to the upper one. All three layers are fabricated from standard 400 µm-thick single-crystal silicon wafers.

This symmetric stacking configuration serves two purposes. Mechanically, it increases the effective proof mass relative to a single-layer design. Geometrically, the mirror symmetry about the mid-plane compensates, to the first order, for the trapezoidal cross-section caused by sidewall tilting during deep reactive ion etching (DRIE) [27,28]. In an asymmetric design, such tilting displaces the effective center of gravity away from the beam plane and can generate a net torque under in-plane acceleration. In the triple-layer design, the upper and lower with-beam layers are symmetrically arranged around the middle mass layer, restoring the center of gravity close to the mid-plane and reducing rotation-related cross-axis coupling.

The sensitive-axis motion can be represented by an equivalent mass-spring-damper system. Let *y* denote the proof-mass displacement along the sensitive *y*-axis and *a_y_* denote the applied acceleration. The dynamic equation is

(1) $$M_{eq}\ddot{y}+C_{eq}\dot{y}+K_{eq}y=-M_{eq}a_{y}$$

where *M*_eq_, *C*_eq_, and *K*_eq_ are the equivalent moving mass, damping coefficient, and stiffness of the sensitive mode, respectively. Under the quasi-static condition used in the tilt-table calibration, the inertial and damping terms are negligible; thus, the mechanical sensitivity *S_m_* and the first-order resonant frequency *f*_0_ can be expressed as

(2) $$S_{m}=\frac{y}{a_{y}}=\frac{M_{eq}}{K_{eq}}$$

(3) $$f_{0}=\frac{1}{2\pi }\sqrt{\frac{K_{eq}}{M_{eq}}}$$

For a stacked structure containing *N_m_* moving mass layers and *N_b_* with-beam layers, the first-order scaling can be written as

(4) $$M_{eq}\;\approx \;N_{m}m_{0},\;K_{eq}\;\approx \;N_{b}k_{0},\;S_{m}\;\approx \;\frac{N_{m}}{N_{b}}\times \frac{m_{0}}{k_{0}}$$

where *m*_0_ and *k*_0_ represent the effective mass of one silicon proof-mass layer and the effective stiffness of one with-beam layer. This relation shows that adding proof-mass layers increases the static sensitivity, whereas adding supporting beam layers increases the equivalent stiffness.

Equation (4) can be used to compare the three layer configurations explicitly. Taking the single-layer structure as *N_m_* = 1 and *N_b_* = 1 gives a normalized sensitivity factor of 1, an asymmetric double-layer structure with *N_m_* = 2 and *N_b_* = 1 gives a factor of 2, and the symmetric triple-layer structure with *N_m_* = 3 and *N_b_* = 2 gives a factor of 1.5. Thus, the double-layer structure is expected to exhibit higher static sensitivity than the triple-layer structure, whereas the triple-layer structure intentionally sacrifices part of this sensitivity gain to restore mirror symmetry and suppress rotation-related cross-axis coupling. The resulting comparison is summarized in Table 1.

The equivalent acceleration noise associated with mechanical–thermal noise can be expressed as

(5) $$a_{n}=\sqrt{\frac{4k_{B}T\omega_{0}}{M_{eq}Q}}$$

where *k*_B_ is Boltzmann’s constant, *T* is the absolute temperature, *ω*_0_ = 2π*f*_0_, and *Q* is the quality factor. This expression indicates that proof-mass enhancement is beneficial for reducing mechanical–thermal acceleration noise, provided that the stiffness and resonant-frequency requirements are simultaneously satisfied.

Because *Q* is strongly affected by the surrounding pressure and package-level damping, the quality factor discussed in this work is treated as an apparent quality factor of the packaged device under the characterization condition, rather than as an intrinsic vacuum quality factor. For the frequency–domain data, *Q*_app_ was estimated from the resonance peak using the half-power bandwidth relation *Q*_app_ = *f*_0_/(*f*_2_ − *f*_1_), where *f*_1_ and *f*_2_ are the lower and upper −3 dB frequencies around the measured resonance.

The cross-axis response may appear as either parasitic translational coupling or parasitic rotational coupling. Acceleration components along non-sensitive axes may induce undesired translational displacement along the sensitive *y*-axis; the *z*-axis component is the representative translational case considered here, although it is expected to be nearly zero under normal vertical mounting. In contrast, in-plane *x*-axis excitation may generate parasitic rotation around the *y*-axis. Therefore, the *z*-axis-induced parasitic *y*-axis displacement and the *x*-axis-induced parasitic rotation are reported separately as translational and rotational cross-axis metrics, respectively.

Cross-axis coupling caused by fabrication-induced sidewall tilt can be described by the eccentricity *e* between the mass centroid *z_m_* and the elastic center *z_k_*. Under cross-axis acceleration *a_c_*, the resulting parasitic moment *M_c_* and parasitic rotation *θ_c_* approximately satisfy

(6) $$M_{c}\;\approx \;M_{eq}a_{c}e,\;e=z_{m}-z_{k},\;K_{\theta }\theta_{c}\;\approx \;M_{c}$$

where *K_θ_* is the equivalent rotational stiffness. In the asymmetric double-layer structure, *e* is nonzero because the mass and beam distributions are not mirror-symmetric. In the triple-layer structure, the upper and lower with-beam layers are symmetrically arranged around the middle without-beam layer, driving *e* toward zero and thereby suppressing *θ_c_*.

The lumped cross-axis relation also clarifies why the symmetric triple-layer design is not selected solely for sensitivity. The asymmetric double-layer configuration benefits from a larger mass-to-stiffness ratio, but its nonzero eccentricity *e* increases the risk of parasitic rotation under in-plane cross-axis acceleration. By contrast, the triple-layer configuration reduces *e* through mirror-symmetric stacking, making it more favorable when sensitivity, structural symmetry, and rotational cross-axis robustness are considered together.

Accordingly, the asymmetric double-layer configuration maximizes the mass-to-stiffness ratio, whereas the symmetric triple-layer configuration trades part of the static sensitivity for improved mirror symmetry and reduced rotation-related cross-axis coupling.

The optical readout converts proof-mass displacement into voltage according to the calibrated optical scale factor

(7) $$\Delta V=G_{o}y$$

where *G_o_* = 0.07 V/µm, as calibrated from the displacement–voltage curve in Figure 1d.

### 2.2. Freeform Geometry Beam Design and Finite-Element Analysis

The upper and lower with-beam layers feature a mirror-symmetric layout, including the sensitive proof mass, connecting rods, freeform geometry beams, stopper structures, and frame. Four identical freeform geometry beams, symmetrically distributed on both sides of the connecting rods, provide the primary elastic suspension. Connecting rods are placed on the top and bottom sides of the proof mass and link it to the freeform geometry beams to form a multi-beam support structure. Stopper structures are arranged on the left and right sides of the proof mass to restrict displacement along the non-sensitive *x*-axis.

The middle without-beam layer contains the proof mass, connecting rods, stopper structures, frame, and shutter rod. The proof-mass shape is consistent with that in the with-beam layers. The stopper structures, together with the frame, restrict proof-mass displacement in the y and z directions, and a local opening is retained at the shutter rod to allow motion within the optical path.

**Figure 2:**
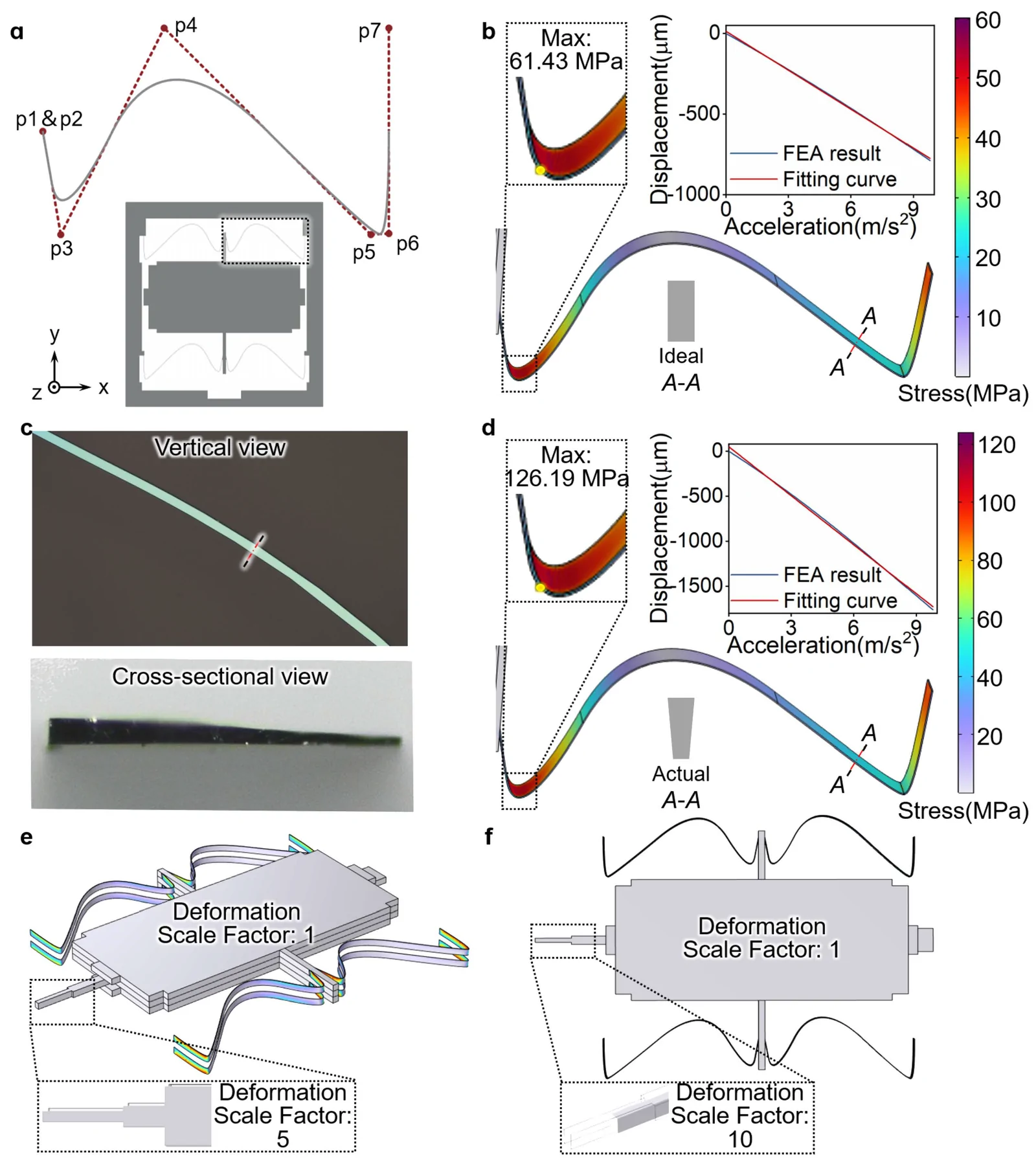
Sensing element optimization results. (**a**) With-beam layer layout with labeled control points P1–P7; (**b**) finite-element simulation results of the triple-layer structure based on the ideal beam structure; (**c**) photograph of the actual beam cross-section; (**d**) finite-element simulation results of the triple-layer structure based on the actual beam structure; (**e**) *z*-axis cross-axis deformation simulation; (**f**) *x*-axis cross-axis deformation simulation.

Given the fixed proof-mass area and predetermined dimensions of the connecting rods, stoppers, and shutter rod, the mechanical performance of the sensing element is predominantly governed by the freeform geometry beams [29]. The beam shape was parameterized based on a quadratic B-spline (order 3) curve defined by seven control points (*P*_1_–*P*_7_), as shown in Figure 2a, and subsequently thickened to a uniform width. Control points *P*_1_ and *P*_2_ coincide and are fixed to ensure a stable connection between the beam and the frame. Control points *P*_6_ and *P*_7_ coincide and are fixed at the central position to connect the beam to the proof mass. The remaining control points define the freeform beam trajectory within the available design region. The coincident endpoint pairs *P*_1_/*P*_2_ and *P*_6_/*P*_7_ are labeled in Figure 2a, and the coordinate ranges and optimized values of all seven control points are listed in Table 2.

The optimization objective was to maximize proof-mass displacement within the 0–1 g acceleration range subject to the permissible stress of silicon and a structural-linearity constraint. The Adaptive Elite Learning Particle Swarm Optimization (AELPSO) algorithm [30] was employed for optimization. Finite-element simulations were conducted using COMSOL Multiphysics 6.3 (COMSOL AB, Stockholm, Sweden), and the optimization was implemented using a coupled MATLAB R2024a–COMSOL procedure.

Finite-element analysis was used to evaluate the mechanical response and cross-axis behavior. Simulations based on the ideal beam structure and the actual sidewall geometry were compared to assess the influence of DRIE sidewall tilting [31]. The optimized triple-layer structure exhibited a displacement of 1.760 mm under 1 g acceleration along the sensitive axis and a first-order resonant frequency of 11.40 Hz. The maximum stress was 61.43 MPa, located at the bend near the connecting rod, which is below the fracture-strength limit of silicon.

The tolerance-included finite-element model was constructed by replacing the ideal vertical sidewall with the measured trapezoidal beam cross-section obtained from cross-sectional inspection, including the sidewall tilt and the associated thickness-direction variation in the effective beam width. These measured DRIE-induced geometry deviations were introduced directly into the COMSOL geometry as deterministic updates, rather than as fitted correction factors, and the resulting sensitivity and resonant frequency were compared with the measured prototype.

The relevant geometric values were extracted from the fabricated beam cross-section shown in Figure 2c and propagated by rebuilding the beam cross-section in COMSOL. The measured sidewall angle was approximately 88.53° according to the angular convention shown in Figure 2c, and the corresponding top and bottom beam widths were approximately 32.81 µm and 12.31 µm, respectively. The in-plane control-point coordinates in Table 2, the 400 µm silicon thickness, and the material properties were kept unchanged. Thus, the tolerance-included result represents a deterministic measured-geometry model.

Under *z*-axis excitation, the parasitic *y*-axis displacement of the triple-layer model was 31.76 µm, lower than that of the asymmetric double-layer model but higher than the ideally symmetric single-layer model. This *z*-axis-induced translation is therefore treated as a translational cross-axis metric. Under *x*-axis excitation, the in-plane rotational response of the triple-layer design was reduced by more than two orders of magnitude compared with the single-layer case and by approximately three orders of magnitude compared with the double-layer case. This result supports the role of mirror symmetry in suppressing rotational cross-axis coupling.

### 2.3. Fabrication, Wafer-Level Stacking, and Optical Readout Assembly

The essential fabrication and stacking process is summarized here because it directly determines the device architecture and optical-readout assembly. The detailed process-level optimization, including the hybrid wet/dry etching strategy, low-temperature adhesive bonding, stepped curing, and minimal-pressure stacking route, has been reported in the related process-focused study [26]. We use this process as the fabrication basis for device-level accelerometer modeling and characterization.

**Figure 3:**
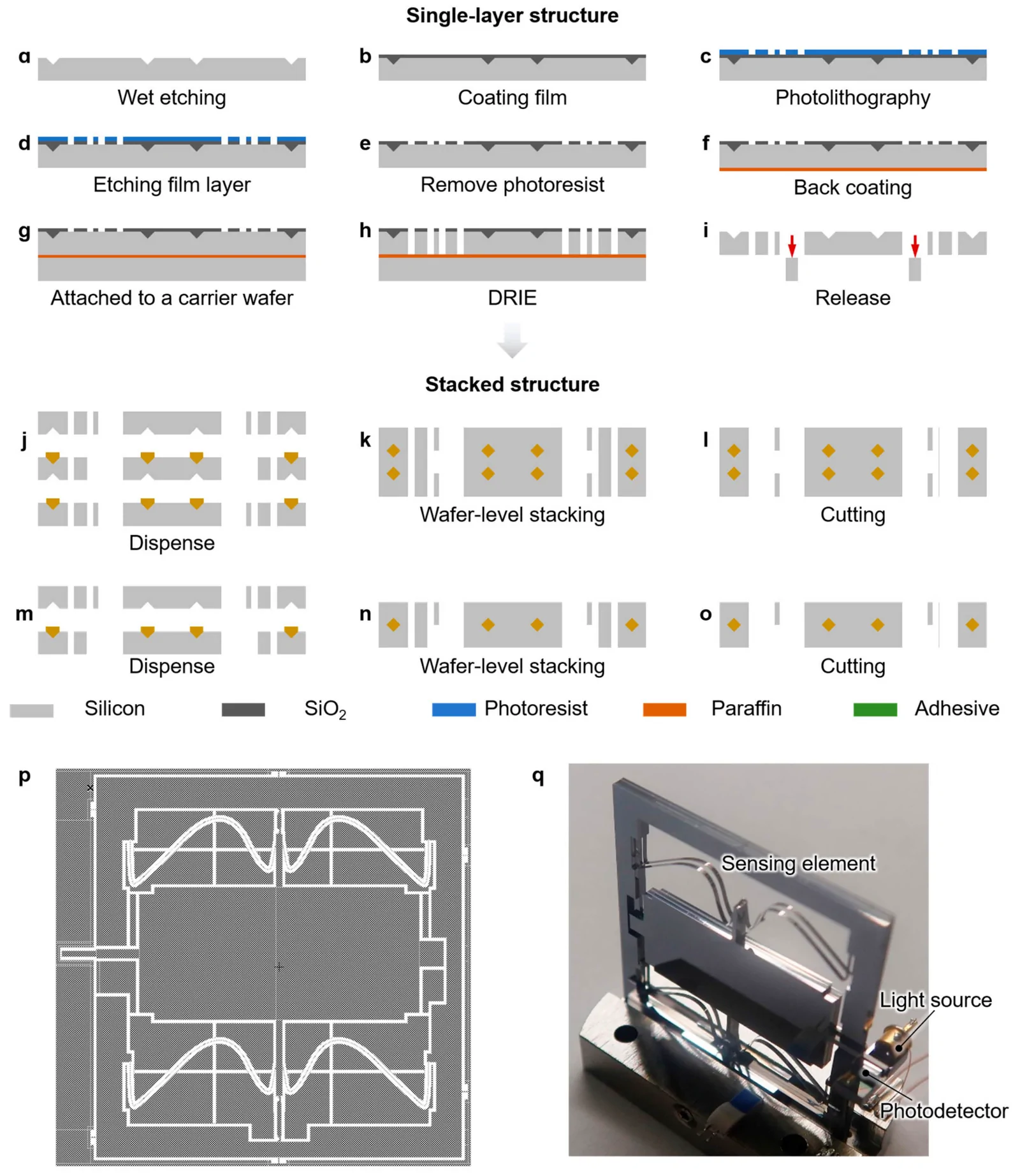
Sensing element fabrication process flow. (**a**–**i**) Single silicon layer fabrication process; (**j**–**l**) structural stacking and assembly process for the triple-layer structure; (**m**–**o**) structural stacking and assembly process for the double-layer structure; (**p**) halo-assisted structural mask layout; (**q**) photograph of the sensing element.

The fabrication flow of one silicon layer is shown in Figure 3a–i. The process combines wet etching and DRIE to form high-aspect-ratio movable structures while maintaining the required sidewall quality. Figure 3j–l illustrates the triple-layer structural stacking and assembly process, while Figure 3m–o shows the corresponding double-layer comparator. A halo-assisted structural mask layout is used to improve etching uniformity across the large-area proof-mass regions. Figure 3q shows a photograph of a fabricated triple-layer sensing element.

Instead of high-temperature direct silicon bonding, a low-temperature adhesive bonding process based on a self-developed composite adhesive was adopted. The adhesive comprises an epoxy resin matrix, an imidazole-based curing agent, and calcium carbonate filler. A stepped curing protocol requiring only minimal applied pressure was used to reduce thermal-mechanical loading at the bonded interfaces. The process is summarized here to support device-level interpretation; the detailed bonding-process characterization is available in [26].

**Figure 4:**
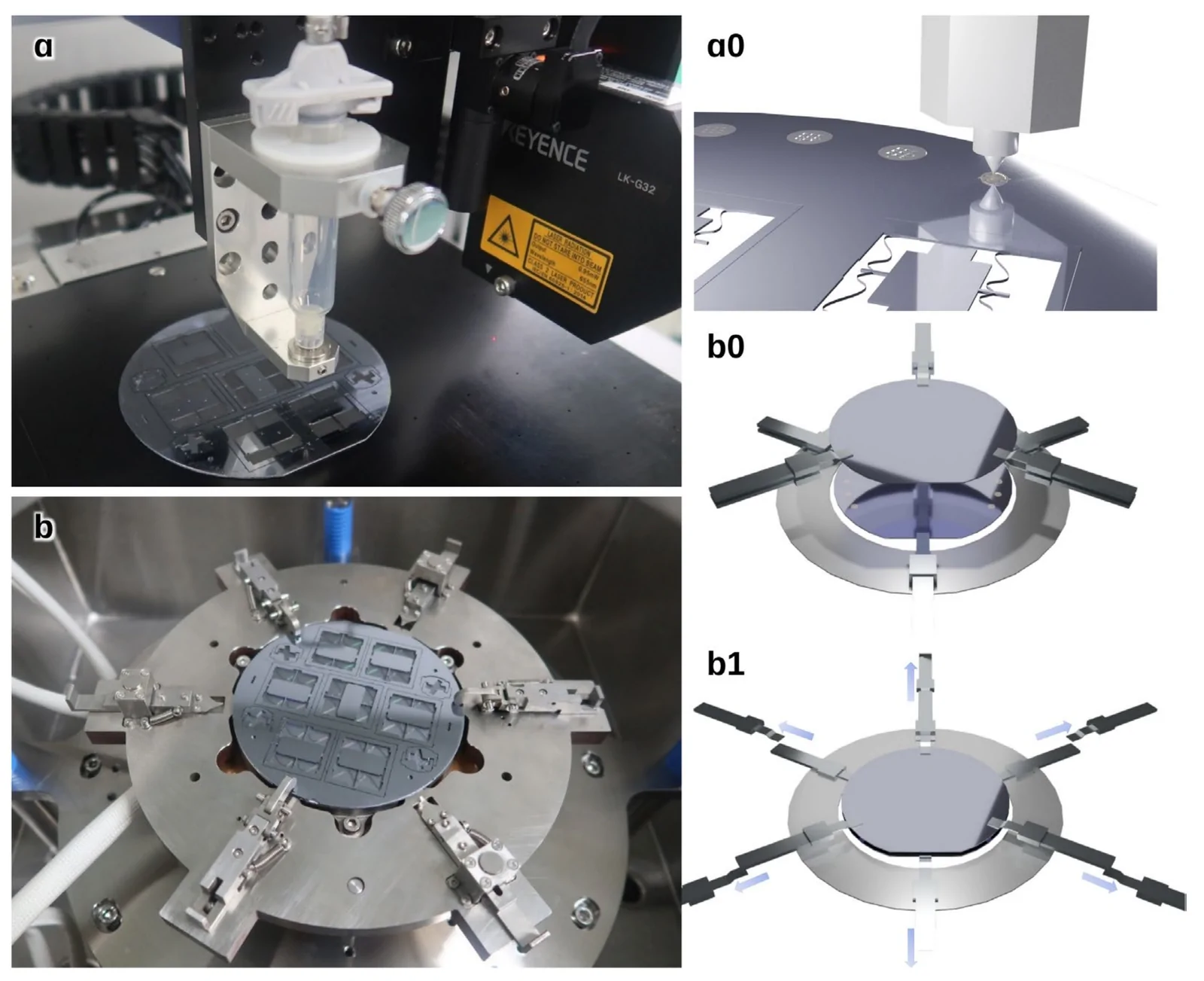
Low-temperature adhesive stacking and alignment technology. (**a**) Photograph of the self-developed automatic dispensing system; (**a0**) schematic of the dispensing process; (**b**) photograph of the self-developed alignment and clamping system; (**b0**) schematic of wafer alignment; (**b1**) schematic of wafer clamping.

Wafer-level assembly was performed using two custom-built systems. An automatic dispensing system deposits the adhesive into the reservoirs with sub-nanoliter volume control (Figure 4(a,a0)). An alignment and clamping fixture with precision alignment pins aligns the three wafers simultaneously (Figure 4(b,b0)) and clamps them during curing (Figure 4(b1)). After curing, the bonded stack is diced and released to yield individual sensing elements.

## 3. Results

### 3.1. Test Setup and Mechanical Sensitivity Calibration

**Figure 5:**
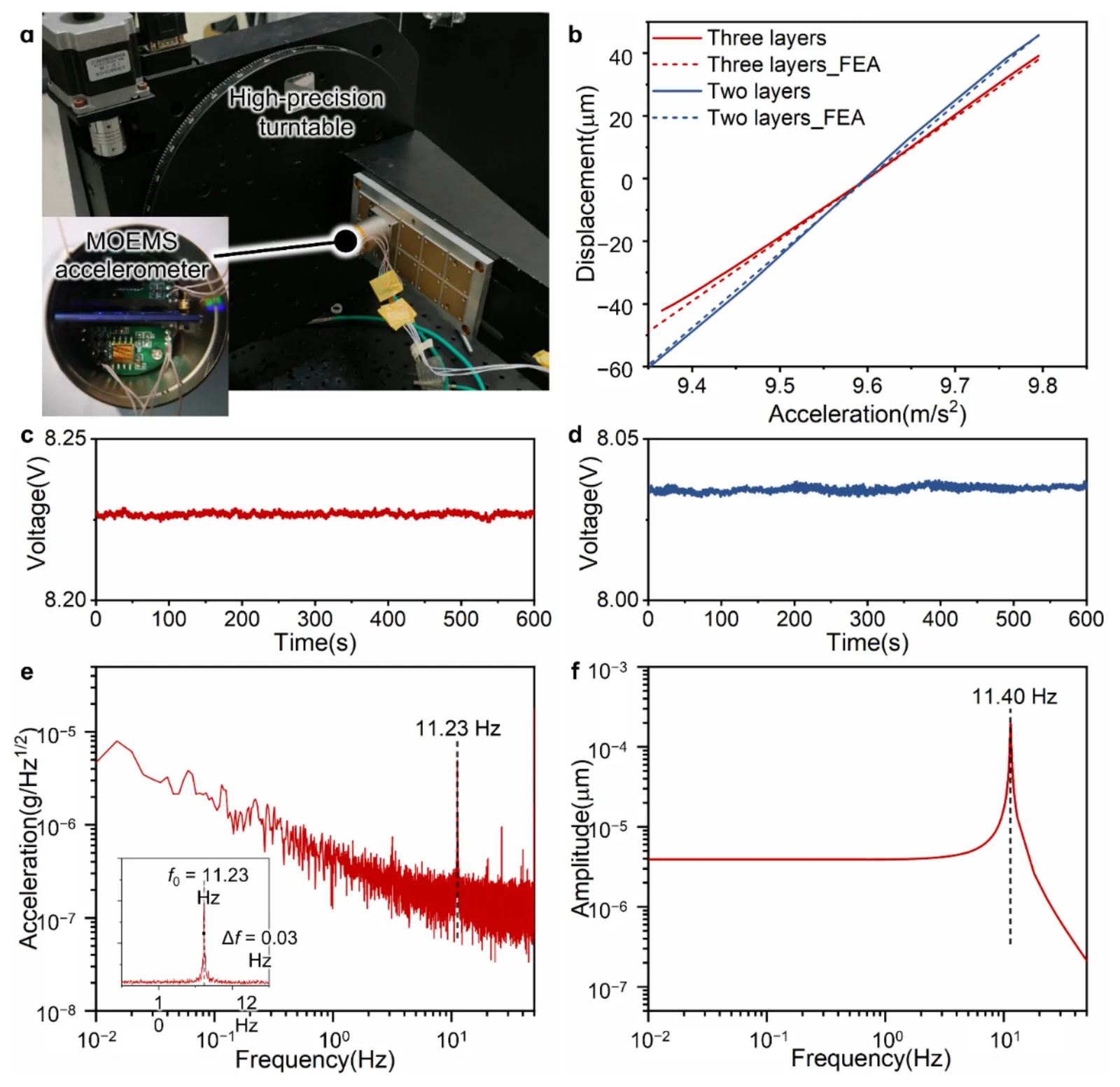
MOEMS accelerometer testing. (**a**) Photograph of the MOEMS accelerometer and experimental setup; (**b**) simulated and experimental mechanical sensitivity results; (**c**,**d**) 10 min output records of the triple-layer and double-layer MOEMS accelerometers, respectively; (**e**) measured acceleration–noise amplitude spectrum of the triple-layer accelerometer showing a first-order resonant peak at 11.23 Hz; (**f**) tolerance-included finite-element dynamic simulation of the triple-layer structure showing a first-order resonant frequency of 11.40 Hz.

The assembled sensing element was integrated with the optical readout module and signal-conditioning circuit and then packaged in a metal housing, as shown in Figure 5a. During assembly, high-precision fixtures and micro-adjustment mechanisms were used to position the shutter rod close to the center of the light beam under a 1 g load along the sensitive axis. This alignment enables nearly symmetric light-intensity reception on the upper and lower detector windows and optimizes the measurement sensitivity and linear operating point.

To evaluate mechanical sensitivity and linearity, the packaged accelerometer was mounted on a high-precision rotary table with its sensitive axis parallel to the table surface. By rotating the table through an angle θ, the gravitational acceleration component along the sensitive axis was varied as g·cos θ. Figure 5b presents the measured responses for the double-layer and triple-layer accelerometers. The measured mechanical sensitivities of the double-layer and triple-layer prototypes were 239.25 µm/(m/s^2^) and 193.91 µm/(m/s^2^), corresponding to 2347.04 µm/g and 1902.26 µm/g, respectively.

The higher sensitivity of the double-layer device is consistent with the lumped-parameter model in Section 2.1. The double-layer structure uses one with-beam layer to suspend two mass layers, whereas the triple-layer structure uses two symmetrically arranged with-beam layers to suspend three mass layers. According to *S_m_* ≈ (*N_m_*/*N_b_*)(*m*_0_/*k*_0_), the normalized sensitivity is 2 for the double-layer structure and 1.5 for the triple-layer structure. The idealized sensitivity ratio of 2/1.5 = 1.33 agrees with the simulated ratio of 2375.89/1760.41 = 1.35 and is also consistent with the measured ratio of 239.25/193.91 = 1.23. For comparison, a symmetric two-layer configuration with two mass layers and two beam layers would have a normalized sensitivity close to that of the single-layer structure. The triple-layer configuration therefore retains a larger mass-to-stiffness ratio while restoring mirror symmetry.

The remaining deviation among the model, simulation, and experiment is attributed to the first-order nature of Equation (4). The ideal sensitivity ratio assumes an identical layer mass *m*_0_, an identical beam stiffness *k*_0_, perfectly uniform bonding gaps, and an unchanged optical scale factor. In practice, the DRIE sidewall taper, local beam-width variation, adhesive-layer thickness, slight optical-alignment differences, and calibration uncertainty can alter both the effective stiffness and the displacement-to-voltage conversion. Therefore, the +8% deviation of the measured triple-layer sensitivity from the tolerance-included finite-element value and the difference between the measured ratio of 1.23 and the idealized ratio of 1.33 are considered reasonable for a single packaged prototype.

### 3.2. Short-Record Output and Dynamic Consistency

Short-record output fluctuation was characterized by placing one packaged triple-layer prototype and one packaged double-layer prototype on a vibration-isolation table. After stabilization, the output voltages were recorded for 600 s at a sampling rate of 10 Hz. No smoothing or filtering was applied to the raw time-series data. Figure 5c,d show the corresponding time–domain output records from the dataset reported in [26]. These records are used here to compare short-record fluctuation between the two stacked configurations.

All short-record output and frequency–domain characterizations were performed at room temperature under ambient atmospheric pressure (approximately 1 atm). The metal package used in this study provided mechanical protection and optical alignment, but it was not a vacuum-sealed package; therefore, the extracted dynamic parameters include air damping and package-level damping.

The 10 min output RMS fluctuation was evaluated relative to the sample mean of each 600 s record. The triple-layer prototype achieved a 10 min output RMS fluctuation of 1.81 µg. In comparison, the double-layer prototype yielded a 10 min output RMS fluctuation of 2.02 µg [26]. In this prototype-level comparison, the triple-layer prototype showed a 10% reduction in the short-record RMS fluctuation. The corresponding noise densities were 0.40 µg/√Hz and 0.62 µg/√Hz at 1 Hz for the triple-layer and double-layer prototypes [26], respectively.

Finally, the dynamic consistency of the triple-layer structure was evaluated using the frequency–domain results in Figure 5e,f. The frequency–domain output in Figure 5e was obtained from a separate static-output recording sampled at 100 Hz. The measured acceleration–noise amplitude spectrum in Figure 5e shows a first-order resonant peak at 11.23 Hz, while the tolerance-included finite-element dynamic simulation in Figure 5f predicts 11.40 Hz. This within-1.5% agreement indicates that the fabricated stacked structure is well captured by the model after fabrication tolerances are considered. Based on the measured magnitude–frequency response, the open-loop bandwidth of the accelerometer was approximately 5 Hz according to the 3 dB criterion, with detailed dynamic-characterization data provided in the Supplementary Materials.

From the measured resonance-peak width around *f*_0_ = 11.23 Hz, the −3 dB bandwidth is Δ*f* = *f*_2_ − *f*_1_ = 0.03 Hz. Therefore, the apparent quality factor is *Q*_app_ = *f*_0_/Δ*f* = 11.23/0.03 ≈ 374.33.

Table 3 compares the present device with representative MEMS/MOEMS accelerometers spanning comparable µg/sub-µg performance levels and related readout strategies, with an emphasis on the sensitivity, bandwidth, dynamic range, readout mechanism, and structural architecture.

As shown in Table 3, the asymmetric double-layer comparator provides higher static mechanical sensitivity, whereas the symmetric triple-layer structure improves the architectural balance by combining mass enhancement with mirror symmetry. Compared with capacitive or resonant MEMS accelerometers, the present device provides high displacement sensitivity and optical-readout compatibility, but its current open-loop bandwidth and experimental cross-axis validation remain limited. Overall, the comparison positions the proposed device as a low-frequency, high-displacement-sensitivity MOEMS platform that prioritizes mirror-symmetric silicon stacking and model-experiment dynamic consistency.

## 4. Discussion

Building on the process-focused wafer-level stacking route reported in [26], this work examines a symmetric triple-layer MOEMS accelerometer that combines mass enhancement, mirror symmetry, and optical displacement readout. The lumped-parameter model explains why the triple-layer design does not necessarily exhibit higher sensitivity than the asymmetric double-layer design, despite its larger proof mass: the additional with-beam layer increases stiffness and partly offsets the mass increase. As summarized in Table 1 and Table 3, the triple-layer configuration should therefore be interpreted as a balanced architecture rather than as the structure with the maximum static sensitivity or the lowest noise floor reported in the broader literature.

Finite-element simulations indicate that the triple-layer design improves the *z*-axis-induced translational cross-axis response relative to the asymmetric double-layer structure and strongly suppresses the more relevant *x*-axis-induced rotational cross-axis response. This separation between translational and rotational cross-axis metrics is important because different non-sensitive-axis accelerations may couple into the sensitive output through different physical pathways.

Cross-axis behavior was evaluated via finite-element analysis using both ideal and measured sidewall geometries. The agreement between the measured and simulated sensitive-axis resonant frequencies provides an independent check of the mechanical model, although direct cross-axis calibration remains necessary for experimental validation of the predicted suppression.

Experimental characterization of one packaged triple-layer prototype shows a measured first-order resonant frequency of 11.23 Hz, in close agreement with the tolerance-included finite-element result of 11.40 Hz. This agreement provides an additional dynamic validation of the fabricated geometry and stacking consistency beyond the quasi-static sensitivity calibration.

The estimated *Q*_app_ also helps interpret the noise relation in Equation (5). For an air-damped packaged prototype with a milligram-level equivalent moving mass, substituting *f*_0_ = 11.23 Hz and *Q*_app_ ≈ 374.33 into Equation (5) gives a mechanical–thermal acceleration–noise floor on the order of 10^−3^ µg/√Hz. This order-of-magnitude value is below the 0.40 µg/√Hz noise-equivalent acceleration reported in [26], indicating that the measured noise is not limited solely by Brownian mechanical noise but also includes optical readout noise, electronics noise, environmental vibration, and slow thermal drift.

For clarity, the main symbols used in the text are summarized in Table 4.

## 5. Conclusions

This article presented the device-level modeling, cross-axis analysis, and optical characterization of a symmetric triple-layer MOEMS accelerometer fabricated using wafer-level low-temperature stacking. The stacked architecture increases the effective proof mass while using mirror symmetry to mitigate the rotation-related cross-axis coupling caused by fabrication-induced asymmetry. A lumped-parameter model clarified the sensitivity trade-off between mass enhancement and added beam stiffness, explaining the measured difference between the asymmetric double-layer and symmetric triple-layer prototypes.

The packaged triple-layer prototype achieved a mechanical sensitivity of 193.91 µm/(m/s^2^), a first-order resonant frequency of 11.23 Hz, and an apparent package-level quality factor of approximately 374. The measured resonance agrees closely with the tolerance-included finite-element prediction of 11.40 Hz. Together with the lumped-parameter analysis and simulation-based cross-axis evaluation, these results demonstrate that symmetric wafer-level stacking provides a practical route to balance proof-mass enhancement, mechanical sensitivity, structural symmetry, and dynamic consistency in MOEMS accelerometers. Future work will focus on direct cross-axis calibration, multi-device statistics, and closed-loop operation.

## Figures and Tables

**Table 1:** Layer configuration comparison derived from the lumped-parameter model and finite-element cross-axis analysis.

Configuration	First-Order Sensitivity Factor	Symmetry and Cross-Axis Implication	Main Advantage	Main Limitation
Single-layer	1	Geometrically symmetric in the layer plane; no stacking-induced mass-centroid offset.	Simple non-stacked structure with no interlayer-alignment error.	Limited mass enhancement; fabrication-induced sidewall asymmetry can still generate rotational coupling.
Asymmetric double-layer	2	One with-beam layer supports two mass layers; sidewall tilt and vertical mass offset can create nonzero eccentricity e.	Highest first-order static sensitivity among the three cases.	Lower mirror symmetry and larger rotation-related cross-axis coupling.
Symmetric triple-layer	1.5	Two identical with-beam layers are placed on both sides of the middle mass layer, reducing eccentricity e and parasitic rotation.	Balanced sensitivity retention, mass enhancement, and rotational cross-axis suppression.	Static sensitivity lower than that of the asymmetric double-layer configuration.

**Table 2:** Freeform geometry beam control-point optimization ranges and results.

Parameter	Definition	Range [µm]	Result [µm]
*x*_1_	Control point *P*_1_ *x*-coordinate	/	0
*x*_2_	Control point *P*_2_ *x*-coordinate	/	0
*x*_3_	Control point *P*_3_ *x*-coordinate	500–3380	500.70
*x*_4_	Control point *P*_4_ *x*-coordinate	3380–6260	3380.90
*x*_5_	Control point *P*_5_ *x*-coordinate	6260–9140	9139.47
*x*_6_	Control point *P*_6_ *x*-coordinate	/	9640
*x*_7_	Control point *P*_7_ *x*-coordinate	/	9640
*y*_1_	Control point *P*_1_ *y*-coordinate	/	0
*y*_2_	Control point *P*_2_ *y*-coordinate	/	0
*y*_3_	Control point *P*_3_ *y*-coordinate	0–2000	297.52
*y*_4_	Control point *P*_4_ *y*-coordinate	0–2000	1804.29
*y*_5_	Control point *P*_5_ *y*-coordinate	0–2000	0.53
*y*_6_	Control point *P*_6_ *y*-coordinate	/	1000
*y*_7_	Control point *P*_7_ *y*-coordinate	/	1000

**Table 3:** Comparison with representative MEMS/MOEMS accelerometers with comparable performance ranges or design objectives.

Work	Device/Readout Strategy	Comparable Performance Parameters	Key Features and Trade-Offs
This work	Symmetric triple-layer MOEMS accelerometer with optical intensity readout	Mechanical sensitivity: 193.91 µm/(m/s^2^); RMS fluctuation: 1.81 µg; NEA: 0.40 µg/√Hz at 1 Hz; Resonant frequency: 11.23 Hz	High mechanical displacement sensitivity; mirror-symmetric wafer-level silicon stacking; close measured/simulated dynamic consistency. Current limitations include open-loop low-frequency operation and the absence of direct experimental cross-axis calibration.
Asymmetric double-layer comparator in this study	Two mass layers suspended by one with-beam layer; optical intensity readout	Mechanical sensitivity: 239.25 µm/(m/s^2^); RMS fluctuation: 2.02 µg; NEA: 0.62 µg/√Hz at 1 Hz; Resonant frequency: ~10 Hz	Higher static sensitivity than the triple-layer structure; non-mirror-symmetric mass/beam distribution and stronger simulated rotation-related cross-axis coupling.
Edalatfar et al. [32]	Capacitive MEMS accelerometer for particle/acoustic acceleration detection	Capacitive sensitivity: ~0.9 pF/g; Noise floor: ~0.6 µg/√Hz; Resonant frequency: 5.2 kHz; Open-loop dynamic range >145 dB	Sub-µg noise with much wider bandwidth at atmospheric pressure; the capacitive high-frequency acoustic target and electrical scale factor are not directly comparable with the present low-frequency displacement-readout architecture.
Barbin et al. [33]	MOEMS accelerometer using an optical tunneling measuring transducer	Sensitivity threshold: ~2 µg; Intrinsic noise: ≤2 µg/√Hz at 1 Hz	Comparable MOEMS/optical accelerometer category with compact optical-tunneling transduction; different non-stacked mechanical and optical architecture.
Wang et al. [34]	MEMS silicon oscillating accelerometer with CMOS frequency readout	Bias instability: 0.4 µg; Noise floor: 1.2 µg/√Hz; Full scale: ±20 g	Low bias instability, large measurement range, and integrated CMOS frequency readout; resonant sensing mechanism and performance priorities differ from the present low-frequency optical displacement architecture.
Gerberding et al. [35]	Optomechanical reference accelerometer	Readout noise: <8 µg/√Hz; High dynamic range and high bandwidth in a vacuum-sealed portable prototype	Reference-grade optical readout with high bandwidth and dynamic range; different reference-instrument architecture and operating environment.

**Table 4:** Symbol definitions used in the main text.

Symbol	Definition
*y*	Proof-mass displacement along the sensitive *y*-axis
*a_y_*	Applied acceleration along the sensitive *y*-axis
*M*_eq_	Equivalent moving mass of the sensitive mode
*C*_eq_	Equivalent damping coefficient of the sensitive mode
*K*_eq_	Equivalent stiffness of the sensitive mode
*S_m_*	Quasi-static mechanical sensitivity
*f*_0_	First-order resonant frequency
*N_m_*	Number of moving mass layers
*N_b_*	Number of with-beam layers
*m*_0_	Effective mass of one silicon proof-mass layer
*k*_0_	Effective stiffness of one with-beam layer
*a_n_*	Equivalent acceleration noise associated with mechanical-thermal noise
*k*_B_	Boltzmann constant
*T*	Absolute temperature
*ω*_0_	Angular resonant frequency, equal to 2π*f*_0_
*Q*	Quality factor
*e*	Eccentricity between the mass centroid and elastic center
*z_m_*	Coordinate of the mass centroid
*z_k_*	Coordinate of the elastic center
*a_c_*	Cross-axis acceleration
*M_c_*	Parasitic moment generated by cross-axis acceleration
*K_θ_*	Equivalent rotational stiffness
*θ_c_*	Parasitic rotation
ΔV	Voltage change of the optical readout
*G_o_*	Calibrated optical scale factor
*N*	Number of samples in the 10 min record
*a_i_*	*i*-th acceleration sample
*ā*	Sample mean of the acceleration record
*σ*_RMS_	10 min output root-mean-square fluctuation

## Data Availability

The data presented in this study are available from the corresponding authors upon reasonable request.

## Supplementary Materials

The following supporting information can be downloaded at: https://www.mdpi.com/article/10.3390/mi17080984/s1, dynamic response and bandwidth characterization of the packaged accelerometer, additional optical-readout calibration data, and supplementary finite-element simulation results.
